# Sex Differences in the Association Between the Systemic Immune‐Inflammation Index and Coronary Lesion Severity in Acute Coronary Syndrome

**DOI:** 10.1002/iid3.70590

**Published:** 2026-11-05

**Authors:** Ming Hong, Chuntian Wang, Hui Liu, Yan Huai, Jiangying Kuang, Huiqiang Chen

**Affiliations:** ^1^ Department of Cardiology The Second Qilu Hospital of Shandong University Jinan China; ^2^ Department of Pediatric Surgery The Second Qilu Hospital of Shandong University Jinan China

**Keywords:** acute coronary syndrome, Gensini Score, sex difference, systemic immune‐inflammation index

## Abstract

**Background:**

Elevated systemic immune‐inflammation index (SII) correlates with increased coronary artery disease (CAD) prevalence and severity in acute coronary syndrome (ACS). The aim of this study was to evaluate the influence of sex on SII and its association with coronary stenosis in ACS.

**Methods:**

A total of 130 patients with ACS, who had performed coronary angiography, were enrolled. Gensini Score (GS) was used to reflect the degree of coronary stenosis. The association of SII and GS were studied.

**Results:**

The mean values of SII and GS were significantly higher (*p* = 0.048 and 0.001, respectively) in male individuals compared with female counterparts. There was a significant correlation between SII and GS (*r* = 0.306, *p* = 0.006) in male participants. However, the positive correlation between SII and GS was not found in female subjects. SII was independently associated with GS (*B* = 0.030, *p* = 0.007) after controlling for age, smoking, and low‐density lipoprotein cholesterol (LDL‐C) in male participants. LDL‐C (*B* = 11.741, *p* = 0.003), smoking (*B* = 39.018, *p* = 0.016) and age (*B* = 1.139, *p* = 0.030), but not SII (*B* = 0.027, *p* = 0.101) were found to be independently correlated with the degree of coronary stenosis in females.

**Conclusions:**

SII was a associated marker for evaluating the extent of coronary lesions in male subjects with ACS, but not in female counterparts.

## 1 Introduction

Despite continuous advances in treatment methods, acute coronary syndrome (ACS) is still one of the leading causes of premature death worldwide nowadays. There is a need to explore the inexpensive and easily available method for disease evaluation and prognosis assessment in patients with ACS. In fact, recent studies have indicated a strong and consistent correlation between ACS and inflammatory parameters [1, 2]. The systemic immune‐inflammation index (SII) is one of such inflammatory markers [3].

SII was developed based on neutrophil, lymphocyte, and platelet and could reflect the balance between host inflammatory and immune response status [4]. More and more evidence has revealed that neutrophils play a major role in not only the initiation and the progression of atherosclerosis but also plaque destabilization and rupture [5]. Research has also shown the vital function of lymphocytes in regulating inflammation throughout the stages of atherosclerosis [6]. In addition, lymphopenia has been linked to the progression of atherosclerosis [6]. The great majority of ACS results from coronary thrombus, which occurs when vulnerable plaque suddenly ruptures, causing platelet aggregation and thrombus formation subsequently in the epicardial coronary artery [7]. A higher level of SII was found to be associated with an elevated level of coronary artery disease(CAD) [8, 9], with more severe CAD [8, 10, 11, 12], and a higher risk of major cardiovascular events (MACEs) and all‐cause mortality in patients with ACS [3] though there are studies with conflicting conclusions [13].

In recent years, it is noted that there are sex differences of inflammatory markers in disease evaluation and prognosis assessment. For example, sex bias was found in the clinical utility of neutrophil, lymphocyte and platelet counts, as biomarkers for predicting prognosis in patients with head and neck squamous cell carcinoma [14]. Cheng et al. found that higher levels of SII were closely associated with an increased risk of all‐cause and cardiovascular mortality in patients with hypertension, in which the association was stronger and more consistent in women [15]. In patients with CAD, it seemed that women had significantly higher platelet counts than men [16, 17]. In males, both neutrophil counts and neutrophil‐to‐lymphocyte ratio (NLR) were higher in patients with CAD than that with non‐CAD [18]. In females, however, there were no differences in either neutrophil counts or NLR in subjects with or without CAD [18]. The above studies raise the possibility that the levels of SII in females are not the same in males in patients with cardiovascular diseases including ACS. Nevertheless, it is not known the influence of sex on SII and its association with coronary stenosis in subjects with ACS.

Here, we aimed to determine sex‐related alteration of SII and then compare the association of SII and coronary artery lesions in male and female patients with ACS.

## 2 Materials and Methods

### 2.1 Study Design and Patient Selection

The study was conducted in accordance with the principles of the Declaration of Helsinki. The institutional ethics committee approved the research proposal. Written informed consent was obtained from all subjects. This cross‐sectional observational study consisted of 130 consecutive participants admitted for coronary angiogram in our center from September 2020 to December 2020 with diagnoses of ACS [19]. Exclusion criteria were coronary artery bypass surgery, coronary artery spasm, malignancies, liver/renal dysfunction, rheumatic diseases, hematological diseases, active infection, history of cardiovascular events, and history of chronic inflammatory diseases.

### 2.2 Data Collection

The demographic and clinical characteristics were collected for each subject including gender, age, body mass index (BMI), as well as history of smoking, hypertension, and diabetes. BMI was calculated by dividing weight in kilograms by height in meters squared. Hypertension was defined as SBP ≥ 140 mmHg, DBP ≥ 90 mmHg, or current use of antihypertensive medication. Diabetes was diagnosed based on the use of insulin or oral hypoglycemic agents.

Venous sampling was drawn in the morning, before coronary angiography and after 12 h overnight fasting. The analysis of blood samples was conducted in the central laboratory of the hospital. The levels of platelets, neutrophil count, lymphocyte count, and white blood cell (WBC) were detected in an automatic detection device. Serum creatinine, fasting glucose, total triglyceride (TG), total cholesterol (TC), and low‐density lipoprotein cholesterol (LDL‐C), high‐density lipoprotein cholesterol (HDL‐C), were detected in an automatic biochemical analyzer in accordance with the standard procedure. SII was calculated via the formula: SII= (platelet count × neutrophil count)/lymphocyte count.

### 2.3 Angiographic Analysis

The selective coronary angiogram was performed by the Judkins technique via the radial artery access. The coronary angiography results were independently evaluated by two cardiovascular experts, who had no knowledge of the patient's clinical data. Gensini scoring system was used to assess coronary artery stenosis [20]. The GS was calculated by assigning a severity score to each coronary artery stenosis based on the severity and geographic significance of the luminal lesions [20, 21].

### 2.4 Statistical Analysis

The Shapiro‐Wilk test was implemented to ascertain the distribution pattern of the continuous variables. Continuous variables with a standard distribution and a non‐standard distribution were presented as mean ± SD or median with 25th and 75th percentiles respectively. The independent Student *t* test was used for the comparison of continuous variables with a standard distribution while the Mann–Whitney *U* test was used for continuous variables with a non‐standard distribution. Discrete variables were expressed as number of cases (n) and percentage (%), and were compared by chi‐square test. The variables with a skewed deviation underwent logarithmic transformation. Correlational analysis was done via Pearson or Spearman correlation coefficient. Multivariate linear regression analyses were performed to explore the correlation between SII and GS in male and female ACS patients. The relevant variables with *p* < 0.05 and traditional cardiovascular risk factors with *p* < 0.1 in the correlation analyses were included in the multivariate linear regression analyses. Statistical analyses used SPSS 23.0 (IBM SPSS, Chicago, IL, USA). *p* < 0.05 were considered statistically significant.

## 3 Results

### 3.1 Clinical and Biochemical Characteristics

A total of 130 ACS patients were enrolled in the study. The median age of the population was 64 years and 62% of the subjects were men. Table 1 presents the clinical and biochemical characteristics of the patients, which were classified by gender.

**Table 1:** Baseline characteristics of the study population.

Variables	Male (*N* = 80)	Female (*N* = 50)	*p* value
Age (year)	62.56 ± 11.40	65.92 ± 8.45	0.057
BMI (kg/m^2^)	25.58 ± 2.68	25.27 ± 3.84	0.620
Smoking (%)	44 (55.0)	4 (8.0)	0.000
Hypertension (%)	61 (76.3)	32 (64.0)	0.132
Diabetes (%)	19 (23.8)	14 (28.0)	0.588
Fasting glucose (mmol/L)	6.12 ± 2.07	5.84 ± 1.36	0.401
Creatinine (μmol/L)	77.24 ± 12.97	60.16 ± 12.32	0.000
TC (mmol/L)	4.11 ± 1.03	4.67 ± 1.37	0.015
LDL‐C (mmol/L)	2.42 ± 0.90	2.78 ± 1.14	0.045
HDL‐C (mmol/L)	0.98 (0.86‐1.15)	1.12 (0.97‐1.25)	0.005
TG (mmol/L)	1.20 (0.79‐1.83)	1.30 (0.88‐1.75)	0.622
WBC (10^9^/L)	7.15 ± 2.07	6.29 ± 1.87	0.018
Neutrophil (10^9^/L)	4.59 ± 1.76	3.80 ± 1.57	0.010
Lymphocyte(10^9^/L)	1.82 ± 0.60	1.85 ± 0.54	0.709
Platelets (10^9^/L)	215.53 ± 48.33	214.40 ± 53.97	0.902
SII	618.91 ± 456.67	476.51 ± 267.77	0.048
GS	67.56 ± 46.04	41.71 ± 34.50	0.001

*Note:* Continuous variables are shown as the mean ± SD or median (25%–75%). Categorical variable is expressed as number of cases (n) and percentage (%).

Abbreviations: BMI, body mass index; GS, Gensini Score; HDL‐C, high‐density lipoprotein cholesterol; LDL‐C, low‐density lipoprotein cholesterol; SII, systemic immune‐inflammation index; TC, total cholesterol; TG, triglyceride; WBC, white blood cell.

As demonstrated in Table 1, male participants had a higher proportion of smokers compared with female subjects (*p *< 0.001). It seems that the male participants were younger than the female counterparts (*p* = 0.057). However, no significant difference was found between the two groups for BMI, hypertension and diabetes rates (*p* > 0.05). The mean values of SII, GS, WBC, neutrophil and creatinine were significantly higher (*p* = 0.048, 0.001, 0.018, 0.010, and < 0.001, respectively) while the circulating levels of TC, LDL‐C and HDL‐C were significantly lower (*p* = 0.015, 0.045, and 0.005, respectively) in male patients compared with female ones. The serum levels of TG, lymphocytes, platelets and fasting glucose did not differ between the two groups (*p* > 0.05).

### 3.2 Correlation Between SII and GS

Table 2 shows the correlation between the degree of coronary artery stenosis, quantified by GS, and the clinical parameters including SII in male and female group (Table 2). As displayed in Table 2, the level of GS was positively correlated with WBC (*r* = 0.242, *p* = 0.031) and neutrophil (*r* = 0.309, *p* = 0.005) in male subjects while the level of GS was positively correlated with TC (*r* = 0.342, *p* = 0.015) and LDL‐C (*r* = 0.369, *p* = 0.008) in female counterpart. There was a significant correlation between SII and the severity of CAD (*r* = 0.306, *p* = 0.006) in male participants. However, the positive correlation between the SII and GS was not found in female patients. Moreover, no significant association were observed between the severity of coronary artery lesions and other cardiovascular risk factors in male or female subjects.

**Table 2:** Correlation between the severity of coronary artery disease and clinical variables including SII.

	GS			
	Male		Female	
Variables	Correlation coefficients	*p* value	Correlation coefficients	*p* value
Age	0.195	0.083	0.267	0.061
BMI	−0.027	0.810	−0.119	0.412
Smoking	−0.060	0.598	0.240	0.093
Hypertension	0.078	0.494	0.010	0.944
Diabetes	−0.060	0.598	0.039	0.790
Fasting glucose	−0.057	0.616	−0.043	0.769
Creatinine	−0.013	0.906	0.096	0.508
TC	0.101	0.371	0.342	0.015
LDL‐C	0.110	0.331	0.369	0.008
HDL‐C(log10)	−0.075	0.511	0.100	0.490
TG (log10)	0.047	0.679	0.035	0.810
WBC	0.242	0.031	0.180	0.211
Neutrophil	0.309	0.005	0.156	0.278
Lymphocyte	−0.115	0.310	0.074	0.610
Platelets	0.002	0.989	0.249	0.081
SII	0.306	0.006	0.134	0.353

Abbreviations: BMI, body mass index; GS, Gensini Score; HDL‐C, high‐density lipoprotein cholesterol; LDL‐C, low‐density lipoprotein cholesterol; SII, systemic immune‐inflammation index; TC, total cholesterol; TG, triglyceride; WBC, white blood cell.

### 3.3 Multiple Linear Regression Analysis

To further explore the association of SII and GS, multivariate linear regression analysis was implemented. The GS was served as the dependent variable while age, smoking, LDL‐C and SII as independent variables. Table 3 and Table 4 presents the results of multivariate linear regression analyses. As displayed in Table 3, SII was independently associated with GS (*B* = 0.030, *p* = 0.007) after controlling for age, smoking, and LDL‐C in male participants. It was LDL‐C (*B* = 11.741, *p* = 0.003), smoking (*B* = 39.018, *p* = 0.016) and age (*B* = 1.139, *p* = 0.030), but not SII (*B* = 0.027, *p* = 0.101), independently associated with the degree of coronary stenosis in female subjects, as shown in Table 4. Thus, SII was independently related to the severity of coronary stenosis specifically in males with ACS, but this relationship was absent in their female counterparts.

**Table 3:** Multiple linear regression analysis with GS as the dependent variable in male.

Variables	*β*	B (95% CI)	*p* value
Age (year)	0.179	0.725 (−0.162 to 1.611)	0.107
Smoking	−0.003	−0.321 (−20.155 to 19.512)	0.974
LDL‐C (mmol/L)	0.176	8.996 (−2.153 to 20.145)	0.112
SII	0.301	0.030 (0.009 to 0.052)	0.007

Abbreviations: CI, confidence interval; GS, Gensini Score; LDL‐C, low‐density lipoprotein cholesterol; SII, systemic immune‐inflammation index.

**Table 4:** Multiple linear regression analysis with GS as the dependent variable in female.

Variables	*β*	B (95% CI)	*p* value
Age (year)	0.279	1.139 (0.116 to 2.162)	0.030
Smoking	0.310	39.018 (7.485 to 70.551)	0.016
LDL‐C (mmol/L)	0.389	11.741 (4.261 to 19.221)	0.003
SII	0.210	0.027 (−0.005 to 0.060)	0.101

Abbreviations: CI, confidence interval; GS, Gensini Score; LDL‐C, low‐density lipoprotein cholesterol; SII, systemic immune‐inflammation index.

## 4 Discussion

In the present study, we showed that SII were positively associated with the extent of coronary lesion in male patients with ACS. In addition, SII could independently identify the severity of coronary stenosis in male subjects with ACS. However, no significant association was found between SII and the extent of coronary artery lesions in female participants with ACS. These results underscore the sex‐related difference of inflammatory markers in evaluating the extent of coronary stenosis in ACS patients.

Atherosclerosis is primarily a systemic inflammatory disease [22, 23]. Inflammation at the site of the atherosclerotic lesion plays a critical role in the pathophysiology of acute plaque rupture in patients with ACS [22, 23]. More and more studies have begun to focus on inflammatory markers and consequently new inflammatory markers have been developed. For example, NLR was developed based on neutrophil and lymphocyte while platelet‐to‐lymphocyte ratio (PLR) was designed according to platelet and lymphocyte. Similarly, SII was exploited on the basis of neutrophil, lymphocyte, and platelet and could well reflect the balance between host inflammatory and immune response status [4]. Recently, it is noted that there are gender differences of inflammatory biomarkers in different age and disease states. For example, the levels of NLR are higher in females than in males in individuals under the age of 50 [5, 24]. The levels of NLR steadily increase in males over the age of 50 while NLR values suddenly decrease around the time of menopause in females [5, 24]. Compared to females, NLR values were significantly higher in male patients with CAD [18], schizophrenia [25], high‐grade glioma [26] and coronavirus disease 2019 [27, 28]. However, platelet values and PLR were lower in male subjects compared with female counterparts [16, 17, 29, 30]. Here, our research revealed that the levels of SII were significantly higher in male patients with ACS than that in female counterparts. It is in line with previous report which showed that SII values were significantly higher in male individuals with high‐grade glioma than those in females [26]. In our study, however, no significant difference was found in platelet values between male and female participants. The discrepancy above could be attributed to the inclusion criteria difference of stable CAD in previous reports and ACS in our study.

In recent years, accumulating evidence indicates a strong and consistent correlation between inflammatory markers and coronary artery lesions [1, 2]. Recent research has revealed that SII was closely related to the risk of CAD [8, 9]. In patients with diabetes or prediabetes, SII could be used to predict the occurrence of ACS in subjects with chest pain [31]. Besides CAD risk, previous studies also indicated that a higher level of SII was found to be associated with more severe CAD in subjects with chronic coronary syndrome or ACS [8, 10, 11, 12]. Recently, Wang et al. reported that SII, which integrates comprehensive clinical characteristics and the systemic inflammatory response index, could predict the occurrence of coronary artery plaque rupture [32]. A higher level of SII was indictive of MACEs and all‐cause mortality in patients with ACS [3] though there are studies with conflicting conclusions [13]. It should be note that the above research studied men and women together and did not study them separately. We here found that SII values were positively associated with the extent of coronary artery lesions and also could independently identify severe coronary lesions in male subjects with ACS, which is in line with the results from overall population. In this regard, SII could be used as a valid parameter for evaluating the degree of coronary artery stenosis in male subjects with ACS. Given that SII is inexpensive and clinically easy to measure repeatedly, it might be used in the risk stratification and prognostic models in male subjects with ACS.

Sex‐related discrepancies exist in the causes, development, and diagnosis of CAD [33]. One unanticipated result of our study was that SII was not an associated marker for identifying the severity of coronary stenosis in female individuals with ACS. It is in line with our previous study, which revealed that the neutrophil to HDL‐C ratio (NHR) was an effective parameter for indicative of severe coronary artery lesions in men, but not in women [34]. Besides SII and NHR, both NLR [18] and the monocyte count to HDL‐C ratio (MHR) [16] were also found not related to the extent of coronary artery lesions in female subjects with CAD. On average, women develop cardiovascular disease a decade later than men. This could be mainly attributed to the protective effects of estrogen [35]. In fact, female sex hormones can directly regulate processes in cardiovascular tissue and indirectly influence disease progression by affecting cardiovascular risk factors [36]. It should be note that the value of hematological biomarkers in subjects with ACS is affected by the health disorders such as diabetes, hypertension, dyslipidemia, anemia, thyroid dysfunction, thrombocytopenia, and chronic renal failure [37]. In response to female sex hormones, delayed neutrophil apoptosis and reduced lymphocyte production have been observed in the bone marrow [38]. In estrogen‐depleted postmenopausal women, the protective effects of estrogen disappears [35]. As mentioned above, we found that female subjects showed significantly reduced levels of SII compared to men with ACS. It should be note that a close relationship was determined between SII and serum vitamin D in patients with ACS [39]. Interestingly, women, unlike men, are more likely to have hypovitaminosis D, which is associated with severe CAD [17]. In addition to conventional risk factors, women are vulnerable to sex‐specific and often undiagnosed cardiovascular issues, including pregnancy‐induced diabetes, preeclampsia, polycystic ovary syndrome, autoimmune diseases, anxiety, depression, and cardiotoxic treatments for breast cancer [40]. The lack of a difference in the female patient group might also be influenced by other confounding factors, such as inflammatory comorbidities, medication use before admission, or ACS subtype. Thus, we speculate that the course of atherosclerosis and ACS in women is different from men and it could not be reflected properly by inflammatory parameters. However, further randomized controlled studies are needed to confirm this.

## 5 Limitations

The present study has several limitations. Firstly, it was a retrospective and single‐center study, which introduces the possibility of center selection bias. The lack of significant differences in female patients may be related to the small sample size. The findings require validation through studies with a larger sample size. Heterogeneity in the medications used by the enrolled patients may also influence the study results. Secondly, the initial results on admission were used to calculate the values of SII, with no consideration given to their dynamic changes during the follow‐up period. Thirdly, the cross‐sectional feature of this study means that long‐term tracking data are lacking, which could not make causal inferences.

## 6 Conclusions

To sum up, SII was a good parameter for evaluating the extent of coronary lesions in male individuals with ACS but not in female counterparts. Future research is needed to examine whether SII could improve the performance of existing risk assessment and prognostic models for ACS in males. The dual nature of SII in males and females should be considered sincerely when clinical assessment and risk stratification of subjects with ACS.

## Author Contributions

**Ming Hong:** data curation, formal analysis, methodology, writing – original draft, writing – review and editing, project administration, investigation. **Chuntian Wang:** methodology, investigation, data curation, formal analysis, project administration, writing – review and editing, writing – original draft. **Hui Liu:** data curation, formal analysis, methodology, investigation, writing – review and editing, project administration, software. **Yan Huai:** methodology, software, investigation, formal analysis, project administration, writing – review and editing. **Jiangying Kuang:** methodology, investigation, software, formal analysis, project administration, writing – review and editing. **Huiqiang Chen:** conceptualization, funding acquisition, supervision, data curation, formal analysis, methodology, software, writing – review and editing, writing – original draft, validation, visualization, resources.

## Ethics Statement

The research was approved by the ethics committee of Qilu Second Hospital of Shandong University.

## Conflicts of Interest

The authors declare no conflicts of interest.

## Supporting information


Supporting File

## Data Availability

Data are available from the corresponding author upon reasonable request.
